# Grounded understanding of abstract concepts: The case of STEM learning

**DOI:** 10.1186/s41235-016-0046-z

**Published:** 2017-01-30

**Authors:** Justin C. Hayes, David J. M. Kraemer

**Affiliations:** 1 Department of Psychological and Brain Sciences, Dartmouth College, Moore Hall 3 Maynard St., Hanover, NH 03755 USA; 2 Department of Education, Dartmouth College, 5 Maynard St., Hanover, NH 03755 USA

**Keywords:** Cognitive Style, Abstract Concept, Conceptual Knowledge, Predictive Code, Hebbian Learning

## Abstract

Characterizing the neural implementation of abstract conceptual representations has long been a contentious topic in cognitive science. At the heart of the debate is whether the “sensorimotor” machinery of the brain plays a central role in representing concepts, or whether the involvement of these perceptual and motor regions is merely peripheral or epiphenomenal. The domain of science, technology, engineering, and mathematics (STEM) learning provides an important proving ground for sensorimotor (or grounded) theories of cognition, as concepts in science and engineering courses are often taught through laboratory-based and other hands-on methodologies. In this review of the literature, we examine evidence suggesting that sensorimotor processes strengthen learning associated with the abstract concepts central to STEM pedagogy. After considering how contemporary theories have defined abstraction in the context of semantic knowledge, we propose our own explanation for how body-centered information, as computed in sensorimotor brain regions and visuomotor association cortex, can form a useful foundation upon which to build an understanding of abstract scientific concepts, such as mechanical force. Drawing from theories in cognitive neuroscience, we then explore models elucidating the neural mechanisms involved in grounding intangible concepts, including Hebbian learning, predictive coding, and neuronal recycling. Empirical data on STEM learning through hands-on instruction are considered in light of these neural models. We conclude the review by proposing three distinct ways in which the field of cognitive neuroscience can contribute to STEM learning by bolstering our understanding of how the brain instantiates abstract concepts in an embodied fashion.

## Significance

Increasing academic proficiency in science, technology, engineering, and mathematics (STEM) fields is not only a goal of educators in these disciplines, but also a national priority spurred on by international comparisons revealing that US high school students currently rank 27^th^ in mathematics and 20^th^ in science out of the 34 nations that comprise the Organisation for Economic Co-operation and Development (OECD, 2012). As new technologies have emerged in recent decades that allow for a more detailed exploration of the inner workings of the brain, there appears to be the promise of brain research becoming a useful resource for improving educational outcomes. However, while research on the brain basis of learning and memory has greatly advanced our understanding of brain function, it has not often been clear how this research can translate to the classroom and inform educational practice. A clearer understanding of the neural basis of STEM learning in general, and a precise evaluation of hands-on learning activities in particular, may be able to play a role in developing activities and structured curricula that allow students to grasp certain fundamental STEM concepts. In the present review, we explore the connection between grounded cognition—the notion that knowledge partially relies on neural mechanisms pertaining to sensory and motoric processes—and STEM learning, evaluating several theories describing how the brain supports concept learning and proposing new research avenues awaiting exploration.

## Introduction

Semantic knowledge consists of the non-episodic, conceptual information human beings use to understand the world (Tulving, 1984). One recognizes the objects in his or her environment by performing neural computations enabling access to a vast repository of information. An object’s identity, function, properties, and form reside in this semantic network, in addition to the abstract knowledge required to understand intricate mathematical and scientific theories. Importantly, a distinction must be made between the ways that the term abstract has been used in the literature. For instance, the concept *dog* can, hypothetically, be represented with a prototype that does not refer directly to an actual dog, but instead a statistical aggregation of the most frequently encountered features of all dogs (e.g., Rosch, 1973). In this way, a mental construct that represents a generalizable form, but not a discrete instantiation, of a concrete object can be construed as abstract. The second sense in which the term abstract is often used denotes a concept lacking a tangible referent in the real world (e.g., Paivio, 1965), such as *justice* or *peace*. In the present discussion, we use the term abstract primarily in this second sense, to refer to intangible concepts (e.g., peace, rebellion). Accordingly, we will use the term concrete when referring to concepts such as *dog*, although we proceed with the understanding that it is necessary to generalize or abstract across exemplars of dogs to refer to a common label. In other words, we consider a concept to be concrete if it refers to an object that may be perceived directly in the world, while abstract concepts rely entirely on relational properties between other concepts (e.g., *peace* is an emergent property of a given state of other concepts and their interactions and relationships to each other).

An effort to understand how abstract concepts relating to science and mathematics are most effectively learned is imperative given that the US educational system now ranks 35^th^ in mathematics and 29^th^ in science, compared to other industrialized nations (DeSilver, 2015). Therefore, domestic policy makers and educators alike are concerned with the state of learning in STEM disciplines. It is no surprise, then, that educational researchers are eager to understand both the mechanisms enabling conceptual knowledge to be stored, accessed, and manipulated, and those optimizing the semantic network for increased learning proficiency.

Theories of embodied cognition, or grounded cognition (GC), may play an integral role in the search for a means to improve STEM learning. Adherents of theories related to GC maintain that sensorimotor networks—brain regions (located in sensorimotor cortex and nearby association cortex) that are preferentially responsive to information within a specific sensory modality—play a prominent role in information processing and semantic retrieval (for a recent review of relevant neuroimaging data, see Martin, 2016). Such networks consist of simultaneously activated brain regions representing the properties of a given concept—for example, seeing a tool activates left hemisphere areas including the ventral fusiform cortex, parietal cortex, and ventral premotor cortex (vPMC), regions associated with visual object identification (form, color, shape, etc.), and manipulation, respectively. Additionally, as frontal and parietal sensorimotor activation occurs not only amidst cognitively demanding tasks (e.g., planning for the use of a hammer), but also while individuals passively view images of tools (e.g., Chouinard & Goodale, 2010), it seems likely that such properties relate to the tool concept itself and not only to specific task demands. With these findings and similar findings related to the embodied nature of semantic knowledge (e.g., Goldberg, Perfetti, & Schneider, 2006; Hauk, Johnsrude, & Pulvermüller, 2004) in mind, it is surprising how little is known regarding the influence of embodied processes on STEM learning (Han & Black, 2011; Kontra, Lyons, Fischer, & Beilock, 2015). Whereas a number of investigations (e.g., Freeman et al., 2014; Winstone & Millward, 2012; Zacharia et al., 2015) have had success exploring teaching methods beyond lecturing—enhancing student engagement through reading, writing, group discussion, and virtual laboratories—we aim to elucidate the benefits of hands-on approaches to learning, an important component in the effort to improve STEM pedagogy.

In the present review, we argue that the cognitive science literature has much to glean from future studies considering how the STEM pedagogy benefits from hands-on activities, designed to bolster the conceptual knowledge underlying scientific learning. We begin this discussion by clarifying the definition of grounded cognition, a controversial term than has been applied to several distinct theories, and then direct the discussion to grounded theories of abstract (e.g., lacking a real-world referent) conceptual processing and representation in the human brain. Finally, we explore possible ways that learning interventions based on theories of GC can benefit students in STEM fields by proposing new empirical directions, bolstering the field’s current understanding of the benefits of tactile learning in both live and virtual environments.

## Review

### Divergent theories of semantic processing

The basic premise of grounded (for our purposes we will interchangeably use the terms grounded, embodied, and situated cognition, but see Barsalou, 2008, for a discussion of some important differences) theories of cognition is that the brain, body, and environment comprise a single, dynamic system. Such a system enables thinking organisms to extend cognition beyond the central nervous system, enhancing computational efficiency by taking advantage of primordial cortical processes (e.g., Dehaene & Cohen, 2007). Considering a knowledge system comprised of these three components has important implications: neural organization necessarily influences how the body and environment are perceived, the body sends feedback to the brain and is used as a metric for navigating the environment (e.g., Witt & Proffitt, 2008), and the environment constrains the ways in which complex behaviors may be executed (Chiel & Beer, 1997). Within this framework, context necessarily influences how knowledge is retrieved and subsequently represented (Kiefer & Pulvermüller, 2012; Yee & Thompson-Schill, 2016), given that context (i.e., geographical, situational, spatial, emotional, cognitive) is an integral component of one’s experiences and the associated concepts. Thus, context influences how a given concept manifests across novel situations. For instance, a hammer is a small hand-held tool used to drive nails when considering a household project, and an ornate weapon of war when contemplating the Norse God of Thunder. Substituting the former object in the context of the latter situation would be erroneous—in other words, one unfamiliar with the story of Thor would be making a semantic error when bringing the household tool to mind while reading about the Demigod’s weapon of choice. Yet, to an outsider, this mistaken individual would appear to fully comprehend the sentence “Thor’s weapon of choice was a hammer.” This illustration explains how two people may have a conversation about an object regardless of whether they have the same object in mind. Therefore, according to the GC view, semantic knowledge is inextricably linked to the context in which it is retrieved, yet flexible enough to be socially transmittable.

#### Amodal theories of semantic representation

The nature of the semantic system is a contentious topic. Amodal theorists (e.g., Fodor, 1998; Mahon & Caramazza, 2009) posit that findings typically attributed to GC are epiphenomenal—i.e., not centrally related to the concepts at hand—and that an amodal symbolic system enables concept retrieval. Accordingly, they argue, while sensorimotor activity enhances semantic representations it is not constitutive of conceptual knowledge, but instead results from spreading neural activation following the retrieval of a given concept in an amodal symbolic system. The key distinction here is that the central component of a concept—that which is crucial to truly knowing it—must exist as an amodal symbol, prima facie, in order for the activity to spread to distinct sensorimotor areas following concept activation. Adherents of this type of theory cite examples of category-specific semantic impairments in clinical populations following lesions to the left temporal lobe, illustrating the modality independence of discrepancies in the semantic network. For instance, cases of double-dissociations in patients unable to recognize either animate or inanimate objects, regardless of the presentation modality, provide support for this theory. Given that focal lesions to this so-called semantic hub can disrupt an entire category of semantic content, the argument for a single cortical conceptual system, divided along categorical lines, is compelling; however, this is not the only interpretation of the available patterns of neuropsycholgical evidence. A number of case studies (e.g., Carbonnel, Charnallet, David, & Pellat, 1997; Kemmerer, Rudrauf, Manzel, & Tranel, 2012; McCarthy & Warrington, 1988) challenge the notion that concept categories exist amodally, represented via the interactions of discrete, iconic symbols located in an innate module in MTL.

#### Multimodal theories of semantic representation

Adherents of grounded theories of semantic representation deny the requirement of elementary conceptual representations for semantic models to function properly. Instead, they argue that concept representations are both multimodal and contextually unique, relying on networks distributed throughout the cortex and reconstructed using the features (shape, texture, sound, etc.) and modalities (visual, phonological, tactile, etc.) in which they were acquired (e.g., Allport, 1985; Carbonnel et al., 1997; Farah & McClelland, 1991; Hsu, Frankland, & Thompson-Schill, 2012; Martin, 2016; McCarthy & Warrington, 1988). For instance, McCarthy and colleagues describe the case of patient (T.O.B.) who was unable to recognize the spoken names of animals, yet, when asked to identify photos of animals, provided robust descriptions. Due to the consistency of the patient’s impairment over time, the authors suggest a phonological impairment leading to semantic deficits. Patient E.C., on the other hand, suffered from complete visual agnosia, in addition to difficulties with non-visual knowledge of animals (Carbonnel et al., 1997). When asked to identify animals using verbal cues, E.C. was better at categorizing domestic rather than wild animals—difficulty recognizing the latter, suggest the authors, exemplifies a visual impairment. In other words, his lack of experience interacting with wild animals restricted E.C.’s ability to represent them in modalities other than vision (also see Warrington & Shallice, 1984; Martin, 2007), while domestic animals, frequently encountered in everyday life, provide a wealth of multimodal (e.g., tactile, emotional, and auditory) experiences for the patient to draw from. Martin’s (2016) GRAPES (grounding representations in action, perception, and emotion systems) theory provides additional support for this idea, suggesting that while concepts are organized based on object properties, such properties are dependent upon one’s experiences—i.e., acquired through a specific sensory modality in accord with one’s physiology and environment. As an example, for sighted organisms, shape is represented in occipital cortex given that the property can be most easily extricated from objects in the environment using vision. In other words, although knowledge about an object’s shape is typically acquired using the eyes, it may be acquired through other senses if vision is unable to encode the property, as is the case for those with congenital blindness. Nevertheless, evidence (Kiefer & Pulvermüller, 2012; Ricciardi et al., 2009) suggests that object properties maintain their general location in the brain regardless of the modality in which they were obtained. For example, the congenitally blind represent object properties typically associated with vision, such as form, in the same areas of occipital cortex as sighted individuals.

Consistent with Martin’s theory and other theories of distributed semantic representations, Farah and McClelland (1991) developed a computational model of semantic memory, noting that categorical deficits emerge spontaneously following lesions within this network comprised entirely of modality-specific subcomponents. The authors assert that categorization results from the correlation between the properties of objects belonging to each category—e.g., living versus non-living. Living things, for instance, are represented primarily by visual traits due to their properties (e.g., visually distinct, non-manipulable) while non-living things are represented primarily based on functional traits due to their properties (e.g., highly manipulable). Furthermore, Farah and McClelland’s model demonstrated that non-primary representations (e.g., functional properties of living things) can be impaired following severe damage to the primary modality (e.g., visual properties of living things), and spared if the damage is minimal. This finding suggests that 1) disparate brain regions rely on reciprocal inputs to reach the threshold necessary to retrieve a given conceptual representation, and 2) whether or not a category is impaired depends on the extent to which the primary representational modality is disrupted. Such a notion is consistent with prior work, including Allport’s (1985) thesis, stating that distributed assemblies of neurons firing in distinctive patterns represent conceptual knowledge. Thus, Farah and McClelland’s model, informed by prior neuropsychological findings (McCarthy & Warrington, 1988; Warrington & Shallice, 1984), provides a parsimonious explanation for both categorical and non-categorical impairments of conceptual representation.

#### A hybrid theory of semantic representation

Perhaps pointing to a synthesis of these neuropsychological findings, hybrid theories of grounded cognition based in cognitive neuroscience (Barsalou, 1999, 2008; Kiefer & Pulvermüller, 2012; Pulvermüller, 2013) argue that conceptual representation relies on both sensorimotor and multimodal (amodal) processes. Hypothesizing that distributed neural assemblies (DNAs) (Kiefer & Pulvermüller, 2012; Pulvermüller, 2013) comprise multiple contextually dependent semantic circuits, such theories account for both sensorimotor and abstract knowledge. This notion is consistent with Barsalou’s (1999) Perceptual Symbol Systems (PSS) theory, asserting that multimodal sensorimotor representations preclude the need for amodal symbols—those typically associated with atomistic conceptual theories (e.g., Fodor, 1998). Convergence zones (CZs; e.g., Damasio, 1989), cortical areas where streams of disparate neural traces converge, likely account for the influence that distinct sensorimotor areas exert upon one another (Farah & McClelland, 1991; Pulvermüller, 2013). Accordingly, nerve impulses originating in modality-specific regions and representing concept features (shape, color, etc.), propagate from their point of origin to adjacent regions, eventually synapsing on interneurons receiving input from additional modality-specific neurons. Such neurons not only integrate feature traces into a coherent representation, but also control timing via Hebbian learning mechanisms (simultaneous firing of NMDA receptors) feeding an integrated trace to higher cortical areas, resulting in robust, multimodal conceptual representations (Friston, 2003). Due to the parsimony of a hybrid, grounded/symbolic account of neural processing, we consider such a theory plausible in light of the following observations:

1. There are numerous studies illustrating the role of sensorimotor cortex in conceptual processing (for a review, see Kiefer & Pulvermüller, 2012; Bergen, 2012).

2. Amodal theories offer a narrow view of conceptual content (e.g., “knowing” is often operationalized as “naming”; Mahon & Caramazza, 2009), and there is ample neuropsychological, computational, and neuroimaging evidence that semantic information is multimodal (Allport, 1985; Carbonnel et al., 1997; Farah & McClelland, 1991; Goldberg et al., 2006; McCarthy & Warrington, 1988; for a review, see Martin, 2007).

3. Concepts are context dependent (e.g., Machery, 2009; for reviews, see Connell & Lynott, 2014; Yee & Thompson-Schill, 2016). Thus, a single, inflexible representational module cannot be assumed to exist in the absence of any direct evidence to that effect, given that multiple neural systems are necessary for contributing contextual cues (e.g., visuospatial regions in occipital and parietal cortex for visually coded geographic information). Without such cues, serving to enrich the meaning of a concept and rendering it contextually flexible, a semantic representation is rendered incomplete.

4. In agreement with Dehaene and Cohen’s (2007) Neuronal Recycling Hypothesis, grounding conceptual knowledge in perceptual and motor systems addresses the question of how advanced human intellectual systems, such as language and mathematical reasoning, developed across a relatively short evolutionary time scale (e.g., writing began ~5400 years ago). On the other hand, a dedicated module for semantic processing would require an evolutionarily expensive process unlikely to occur in such a short amount of time.

Thus, the semantic knowledge system appears to entail sensory-specific representations—organized according to within-modality features—relying on convergence zones to integrate information across modalities. So far, however, we have mainly considered data pertaining to knowledge of concrete objects. Given the importance of abstract knowledge for both education and general learning, we now consider how a grounded system supports knowledge of abstract concepts.

### Sensorimotor contributions to abstract concept retrieval

The notion of a concept being abstract can be conceived in two distinct ways (see Introduction). For instance, any concept potentially represented in the mind by a prototype (e.g., one’s mental image of *carrot;* Rosch, 1973) could be characterized as being abstract. Here, we consider a second sense of the term abstract, one consistent with Paivio’s (1965) work, suggesting that nouns exist on a spectrum ranging from concrete to abstract, and that nouns representing concepts referring to perceptible objects evoke the most vivid mental imagery. Conversely, concepts with no tangible referent, such as *peace* and *justice*, are more difficult to process because they do not evoke the same type of mental imagery. Therefore, in the present discussion, we describe intangible concepts as abstract and those more directly accessible to the sensory system as concrete.

To further illustrate this distinction, consider that when one reads words such as *justice* and *compassion* in everyday encounters, such ideas are embedded in a situational context—e.g., a criminal being apprehended by the police or a family rescuing a dog from an animal shelter, respectively. Moreover, contexts vary drastically for abstract concepts given the flexibility of their meaning (Barsalou, 2008; Granito, Scorolli, & Borghi, 2015). One might describe *bravery* in the context of a soldier rescuing a fellow soldier on the battlefield; the term could also describe a shy student giving a speech in front of her peers. While the concept itself is representative of the same central idea across both of these contexts, i.e., carrying out an action despite one’s fears, these two situations share no common perceptual features. Instead, understanding that the concept bravery applies in both situations lies in comprehending the relationship between an agent and his or her context (actions, environment, etc.). Therefore, the underlying commonalities across these unique instantiations of such concepts are understood through analogical reasoning processes (e.g., Gentner, 1983; Gick & Holyoak, 1983). In other words, abstract concepts consist of the relational properties arising from the interaction of two or more objects or agents in a given circumstance, and such concepts share an underlying commonality despite dissimilarity on the surface-level (i.e., perceptual features). The multimodal contextual features of concrete concepts, on the other hand, are relatively more consistent across contexts, providing a great deal of information about the meaning of the concept. For instance, door knobs vary in subtle ways, but they generally maintain a recognizable form and are found within a proscribed area on almost any door. Therefore, we consider the primary distinction between abstract and concrete concepts to be the features comprising their representations—i.e., concrete concepts refer to tangible objects and abstract concepts refer to the emergent properties which result from the interaction of concrete concepts.

The context of abstract semantic content is unstable, while that of concrete semantic content is comparatively more durable—i.e., a carrot possesses diagnostic features that enable its identification despite subtle contextual discrepancies (a carrot may be brown but maintain its form; a carrot may have a different form but maintain its salient orange color, etc.). Therefore, it is more difficult to retrieve the meaning of abstract compared to concrete concepts. This is likely due to both the rapid recall of multimodal features associated with a given concrete object concept, and the computational demands necessary for retrieving the structural similarity between abstract concepts, resulting from unpredictable variation in contextual features across situations. Therefore, when asked to identify an abstract concept, in a word/non-word task for instance, if the concept is presented in isolation with no contextual information, one must construct a context in real time in order to understand the concept—this would explain why individuals are slower to identify abstract concepts (Schwanenflugel & Shoben, 1983; Xiao, Zhao, Zhang, & Guo, 2012).

Additionally, abstract concepts, unlike concrete concepts, in which an object is perceived before being assigned a label, require a label to subsume the contextual constraints associated with the concept. This account is consistent with the notion of Recchia and Jones (2012) that comprehending abstract words requires context-specific cues, while concrete words rely on object features. Namely, when processing words describing concrete concepts (e.g., *car, dog*) in a scene, people tend to focus their attention to the features of the objects themselves; conversely, when learning novel abstract words (e.g., *disorder*), attention is shifted toward the scene as a whole, while individuals attempt to establish the relationship between agents and objects in the display (Granito et al., 2015). Consider an image depicting the Boston Tea Party, when asked to identify the *tea* concept, one might point out the substance being dumped from bags into the harbor; conversely, if asked to identify *rebellion,* one would rely not only on the interaction between agents engaging in rebellious behavior, but also the geographic location, the affect of the agents and bystanders, and biographical knowledge gleaned in a history class. Thus, while identifying a concrete concept requires one to evoke a flexible yet consistent prototype acquired via statistical regularities across contexts (e.g., Kiefer & Pulvermüller, 2012), identifying a concept that is entirely relational requires a great deal more computation resulting from the process of discovering an analogous relationship between contexts, and thus searching a scene for cues denoting such a relationship which is necessary to determine if a given token fits the concept (see the “Predictive coding and Hebbian learning” section below).

Dove (2016) argues that abstract concepts are not easily reconcilable under current embodied theories, citing a number of physiological studies demonstrating activation differences while participants process concrete rather than abstract concepts. Furthermore, familiarity with such concepts results in their apparent dissociation from sensorimotor regions and an increased reliance on left hemispheric structures (see Binder, Westbury, McKiernan, Possing, & Medler, 2005). While such findings may appear to bolster amodal conceptual theories, suggesting distinctive mechanisms for processing concrete and abstract concepts, the shallow recognition protocols (e.g., lexical discrimination tasks) used in many such tasks fail to account for the contextual relativity inherent to abstract knowledge. In other words, recognizing a word does not require the same depth of information processing as retrieving its meaning. Xiao et al. (2012), for instance, argue that contextual details facilitate recollection of concrete words leading to faster RTs during a recognition task. Evidenced by an increased parietal P600 response for tangible concepts, an evoked potential typically associated with the integration of contextual details, the authors conclude that abstract words are more difficult to process given the absence of such details when interpreted independently of context, which is crucial for understanding them (Katja Wiemer-Hastings & Xu, 2005). In other words, concrete concepts (e.g., *car, shoe*) evoke a rapid representation grounded in modality-specific features (visual, tactile, etc.) while abstract concepts (e.g., *inequality*) require external conceptually relevant cues used to derive a complete understanding of the idea.

Thus, understanding abstract concepts requires one to comprehend the relationship between objects, rather than the objects themselves. This is an idea that is entirely consistent with the literature addressing analogical transfer (e.g., Gentner, 1983). Consider the resemblance between what we have characterized above as an abstract concept (e.g., *preparation*) and a typical example of an analogy—one might, for instance, construct an analogy to describe *preparation* by comparing a student studying for a difficult examination to a long-distance runner training for a marathon (law student : studying :: marathon runner : training). Gick and Holyoak (1980, 1983) refer to the perceivable properties typically associated with concrete concepts as *surface features*, while referring to those denoting the underlying relations between two or more terms of an analogy as *structural features*. Therefore, in our example, the two instances of *preparation* do not share surface features as there is no direct mapping between the perceptible features of a student studying for a law examination and a runner training for a marathon. However, the underlying relations between the analogies are consistent—i.e., both runners and students must engage in preparations in order to accomplish their goal. Thus, the structural properties of the two instantiations of *preparation* are shared, while the surface features of the two analogous scenarios are not.

A natural consequence of the human tendency to construct analogies is apparent when considering how metaphors are used to relate everyday notions to broader concepts. Lakoff and Johnson (1980), for instance, argue that almost all human conceptual thought relies on metaphor. Consider, for example, how Westerners conceive of competitive and/or contentious interactions as war-like—rap *battles*, *fighting* disease, culture *warriors*, etc. As Lakoff and Johnson point out, this framework is a cultural artifact, grounding concepts in a familiar, concrete context. Furthermore, assert the authors, such a framework influences how individuals behave—e.g., an argument is seen as an attempt to *defeat* another person’s position; as such, one approaches an argument to leave his or her opponent in a state of *defeat*. Thus, if an alternative, non-quarrelsome metaphor was used to characterize competitive exchanges, the nature of the interactions would change, as agents behave in a manner relative to the underlying framework. For example, consider an argument in the framework of a cooperative game—rather than *striking a blow* to an *opponent’s* argument, one might, instead, *take a turn* in order to *advance toward a common goal*. Similarly, Boroditsky (2011) considers the notion of time within such a culturally derived framework, summarizing several experiments that demonstrate how time is understood relative to culturally and linguistically informed constraints. For instance, time is often mapped onto spatial dimensions which vary according to how a given language describes temporal movement (e.g., time is up/down, forward/backward) and how that language is written (horizontally/vertically), providing an additional means to ground abstract concepts. Native speakers of Mandarin Chinese, for instance, regularly describe time as occurring vertically; English speakers almost exclusively refer to time as if it exists on a horizontal plane, advancing from left to right (Boroditsky & Gaby, 2010). Speakers of both English and Mandarin, therefore, conceive of time in a way that agrees with how their language is written. Thus, the culturally derived metaphors ubiquitous in natural languages may influence how individuals think about their worlds as they map specific concepts onto broader notions grounded in concrete ideas, such as spatial dimensions.

In addition to Lakoff and Johnson’s assertion that a metaphorical framework influences how concepts are understood, a related though contentious literature argues that physical states directly influence how abstract metaphors are understood. Consistent with the Western notion of the trajectory of time, one study found that individuals tended to lean forward when thinking about the future and backward when considering the past (Miles, Nind, & Macrae, 2010). Another study found that individuals who had recently recalled a situation where they were socially excluded guessed that the room they were in was colder than did individuals who had recalled a socially inclusive experience, suggesting that being ostracized (i.e., “treated coldly”) literally evokes a cold sensation (Zhong & Leonardelli, 2008). These outcomes suggest that human beings may understand abstract concepts by mapping them onto concrete objects or physical sensations with which they are able to directly perceive or experience. Barsalou and Weimer-Hastings (2005) suggest that abstract representations rely on internal states to derive meaning across situations—e.g., when seeing or hearing the word *justice* a feeling of strength and relief is experienced in the body. This suggests that abstract verbs are associated with actions and feelings, involving an exchange between one or more agents, and informed by the context—interaction of mental, physical, and environmental cues—in which they occur.

Due to the evidence considered thus far, we would predict that, at the systems level, abstract concepts are associated with distributed neural representations grounded in contextual—social, linguistic, affective, spatial, and sensorimotor—regions of cortex, at least in terms of processing the semantic meaning of the associated terms. Additionally, evidence suggests that the left frontal polar region is a key structure for integrating the structural properties between disparate analogous relationships—i.e., abstract concepts (Bunge, Wendelken, Badre, & Wagner, 2005; Green, Fugelsang, Kraemer, Shamosh, & Dunbar, 2006; Green, Kraemer, Fugelsang, Gray, & Dunbar, 2010). Therefore, we would expect this region to be active when one assesses a given scenario to determine whether or not it fits the criteria of an abstract concept—e.g., is the Boston Tea Party in fact an example of *justice*? Consistent with these ideas, after controlling for resting state and linguistic activity, Wilson-Mendenhall, Simmons, Martin, and Barsalou (2013) found increased activation in neural regions associated with social cognition and mentalizing (medial prefrontal cortex, posterior cingulate, orbital frontal cortex, and superior temporal sulcus) while participants computed the meaning of *convince*, and increased activation in regions associated with mathematical processing (intraparietal sulcus, superior parietal cortex) while participants computed the meaning of *arithmetic.* Importantly, after averaging the brain activity for the two concrete and abstract concepts, these context-specific distributed representations vanished, suggesting that averaging across concepts may misconstrue activation patterns unique to the concepts they represent. These data imply a great deal of variance in the neural foundations of abstract concepts—consistent with the idea that the conceptual representations that form the neural basis of relational abstract ideas are context dependent and supported by dynamic patterns of activity over distributed networks.

#### Predictive coding and Hebbian learning

Evidence conferring a computational advantage for contextually rich concrete concepts (Xiao et al., 2012) adheres to the predictive coding (PC; e.g., Barsalou, 2013; Friston, 2005; Summerfield et al., 2006) account of conceptual retrieval. In essence, predictive coding describes a hierarchical process by which sensory signals sent from bottom-up perceptual systems in the brain converge with top-down signals, or models. Such models are derived from data collected over repeated exposure to a given concept or situation—insofar as models are unable to account for all situational variance, error signals are necessary for updating inaccurate predictions at each stage in the hierarchy. Thus, predictive coding is predicated on the notion that cortico-cortical circuits: 1) are arranged hierarchically and 2) are comprised of feedforward (ascending) and feedback (descending) connections between subcortical structures and cortex, 3) include both driving and modulatory connections, and 4) interact such that cortical neurons are able to model corporeal states and subsequently modulate sensorimotor neurons based on feedback loops producing error at each level in the hierarchy (Friston, 2003). Thus, correlating with incoming sensory data, cortical regions predict external conditions based on models derived from prior experience; this top-down model is compared with perceptual input and updated via modulatory interneurons at each layer in the hierarchy until the model closely matches the sensory data, thereby reducing error in the signal (Friston, 2012).

According to Friston, the predictive coding paradigm aims to minimize entropy (e.g., uncertainty) in the system via statistically aggregated, Bayesian inference models. It is no surprise, then, that concrete knowledge is modeled with greater efficiency than abstract knowledge given its relative stability across contexts. In other words, although concrete object concepts such as *door* can take on many forms, the functionality and central features of a door (e.g., opens and closes, serves to divide two adjacent areas when closed and connect them when open) are consistent. On the other hand, abstract concepts such as *justice* are both context dependent (e.g., one may be considered just or unjust when stealing, depending on the circumstances) and relational (multiple agents are required for an act of justice to take place). Thus, the features correlated with a circumstance in which one might encounter an example of *justice* are not as stable across contexts as those correlated with *door*—I know that when I walk into a new building that I am extremely likely to find a door and I will certainly know how to use it, but whether or not I experience *justice* and how the concept will play out in a given context is radically different from one situation to the next, depending on both subjective judgment (e.g., how unjust is it for a sick individual to avoid paying a medical bill she cannot afford to pay?) and cultural norms (see Borghi & Cimatti, 2009, Boroditsky, 2011). Thus, abstract concepts, compared to concrete-object concepts, cannot be easily captured by a predictive model due to the amount of error inherent in such a model. As a result, a larger computational burden is likely placed on hierarchical cortico-cortical networks while processing abstract concepts, as top-down and bottom-up circuits work to interpret contextual variability.

Abstract concepts rely on data spanning a number of unique circumstances. The word *compassion*, for instance, is not directly correlated with a movement or sensory representation. Nonetheless, the PC model is applicable to this and other concepts, and several theories (e.g., Barsalou & Weimer-Hastings, 2005; Martin, 2016) address this discrepancy. Barsalou and Weimer-Hastings, for instance, contend that abstract concepts are evoked in the presence of an applicable situation; thus, when an individual witnesses a college student helping an elderly man with his groceries, she assigns the label *compassion* to the relationship between agents in a situational context. According to the PC model, a high-level representation of an abstract idea is generated when witnessing an applicable example—an episodic event comprised of social and contextual features which may be referenced when encountering future instantiations of the concept. As incoming sensory data conflict with the model’s expected outcome, the model is updated to accommodate new information. For example, if after helping the elderly man one sees the college student being paid for his assistance, the current model must be updated, as *compassion* does not include selfish motives. Hence, we expect to see an alteration in the neural representation of the concept following this update, as the model is revised. Therefore, while abstract concepts are capable of being modeled within a predictive coding hierarchy, we propose that such models are highly volatile and are thus inconsistent predictors of the semantic features of a given concept.

Similar accounts pervade the literature. For example, theories attributing conceptual knowledge to distributed cell assemblies (e.g., Martin, 2016; Pulvermüller, 2013) propose that disparate sets of neurons representing distinct modality-specific properties (color, form, sound, texture, etc.) aggregate in convergence zones (Damasio, 1989) located near the center, or hub, of neural circuits where divergent regions converge to bind the features of a given conceptual representation. Convergence zones are likely candidates for the high-level conceptual models within the PC framework—low-level feature circuits intersect in CZs where they inform and/or update the current representation based on error signals. Importantly, several studies (e.g., Hsu et al., 2012; Simmons et al., 2007) report real-time feedback between low-level perceptual areas in occipital cortex and higher-level conceptual areas in fusiform gyrus for color perception, providing direct evidence of PC mechanisms.

While PC provides a plausible mechanism for modeling concepts both online and offline, it is also necessary to discuss how divergent streams of information come to be assembled within CZs, producing Bayesian hierarchical models. Hebbian learning (e.g., long-term potentiation; LTP) offers a proven and parsimonious explanation of this phenomenon. According to LTP, when two or more seemingly distinct events occur simultaneously across a number of episodes, the synaptic connections encoding the representation of each event are strengthened to the degree that the neural firing associated with event A is enough to cause firing across the synapse associated with event B and vice versa (Hebb, 1949). Hebbian associations, therefore, are the building blocks of predictive models, as the features of events which typically co-occur are hardwired together across neural circuits. For instance, several papers (Glenberg & Gallese, 2012; Lee, Turkeltaub, Granger, & Rizada, 2012) hypothesize that language production shares a Hebbian association with language comprehension. This relationship may in fact account for language development—as the babbling infant learns to associate specific mouth movements with their correlated sounds, neurons in Broca’s Area form an association with those in auditory cortex. Thus, concurring with Lee and colleagues, novel mouth movements are associated with distinctive speech sounds and, therefore, hearing a word may activate motor areas recruited when speaking the word, leading to an understanding of the word via subsequent activation of associated modality-specific semantic networks. Ibáñez et al. (2013) provide direct evidence for this notion, evaluating the Action-Sentence Compatibility Effect (ACE; see Glenberg & Kaschak, 2002) in epilepsy patients. The ACE task requires patients to respond to a cue by moving in a direction either compatible or incompatible with directional information implied in a sentence (e.g., “John was moving on after the breakup” = forward movement). Previous studies have demonstrated robust interference, as evidenced by slower RTs, when the motion used to respond mismatches that implied in a sentence. Ibáñez et al. (2013) measured the ACE using electro-corticography in two epilepsy patients awaiting surgery by placing subdural electrodes on the surface of the left fronto-temporal and frontal cortex. When measuring both language and motor responses while the patients processed the final verb in a given sentence, a bi-directional effect was observed, evidenced by a negative evoked potential at 400 ms—a correlate of semantic processing—in premotor, motor, and language areas during incompatible trials. Presumably, the increased N400 response, typically associated with an unexpected stimulus (e.g., Fabbri-Destro et al., 2015), indicates incompatibility between the meaning derived from a sentence and the direction of the required response. This outcome suggests that both linguistic and motor content provides meaningful information to readers, as the motor system enhances language understanding while language understanding modulates the motor system.

Consistent with these principles, Barsalou’s (2013) Pattern Completion Inferences within Situated Conceptualizations (PCIwSC) theory suggests that associational learning mechanisms may augment or even replace extant theories of conceptual modeling. PCIwSC proposes that multiple neural networks representing disparate features are integrated to capture the totality of a concept by processing parallel streams of contextual, self-referential, social, and sensorimotor data simultaneously. For instance, according to this model, if I find myself in a restaurant where I am meeting a coworker typically dressed in a suit and he arrives in jogging pants and a t-shirt, it may take me a few extra seconds to recognize him. This delay may be due to the necessity of integrating novel input into and subsequently updating an erroneous model, given the fact that I have come to associate a set of visual features (how my coworker is dressed) with that person. Within my predictive model, for example, the features of the face match my prediction; however, because I am viewing my coworker from across the room, the face prediction is not entirely accurate and his clothing provides a mismatch with my current predictive model.

Thus, Hebbian learning connects the sensory, motor, and affective information constitutive of high-level models. Further, the PC theory provides a parsimonious explanation for concept formation, one which eschews the need for a modality-independent semantic system— a controversial idea that opposes what we know about the timescale for evolutionary development (for more on this, see Barsalou, 2008; Dehaene & Cohen, 2007). It is, therefore, not surprising that familiar objects, locations, and smells are able to evoke elaborate conceptual representations (the carnival or grandma’s house, for instance), given that a single feature is capable of evoking additional features constitutive of the entirety of the concept. Consistent with this notion, widespread bilateral neural activation patterns for both abstract and concrete concepts are associated with faster RTs in a word/non-word task, suggesting that highly distributed representations confer a retrieval advantage (Binder et al., 2005). With this advantage in mind, we propose a grounded theory of STEM learning based on predictive coding and Hebbian learning paradigms.

### Neural representations of STEM concepts

Given the relatively short history of human scientific inquiry, beginning with astronomy less than 10,000 years ago (Ruggles, 1999), and because we know that it takes hundreds of thousands of years for distinct neural systems to develop (Dehaene & Cohen, 2007), it is highly unlikely that the human brain developed a distinct neural system dedicated to processing the type of information central to scientific conceptual understanding. Instead, it is likely that older neural systems have accommodated and influenced the trajectory of scientific thinking. For instance, Dehaene and Cohen (2007) point out the architectonic similarity between left hemispheric regions associated with perceiving natural objects, the fusiform face area (FFA) for example, and the visual word form area (VWFA; thought to specialize in the recognition of written language patterns)—another recently developed, culturally derived skill. These regions neighbor one another and follow a distinctive hierarchical trajectory, starting with cells specializing in perceiving primitive shapes in the occipital cortex, and ending in more anterior regions specialized for perceiving complex forms (e.g., words or faces). Thus, Dehaene and Cohen hypothesize that the human brain has co-opted the existing function of regions that evolved to perform tasks associated with our evolutionary lineage—e.g., recognizing natural objects in the environment. Accordingly, culturally dependent functions, such as word recognition and comprehension of symbolic number magnitude, bootstrap the hardware necessary for more primitive computations, such as object recognition and estimation of physical magnitudes (e.g., size, distance, quantity; but see Lyons, Ansari, & Beilock, 2015).

Supporting this assertion, Mason and Just (2016) used fMRI to map the neural representation of physics concepts in undergraduate and graduate students, and their results were consistent with those of the neuronal recycling theory of Dehaene and Cohen (2007), in addition to theories rooted in grounded cognition and predictive coding. The authors divided physics concepts into four discrete categorical factors—causal motion, periodicity, algebraic equation representation, and energy flow (also controlling for word length as a fifth factor)—discovering that each factor was not only discernable based on activation patterns, but also associated with activation in regions of the cortex underlying primitive processes, such as spatial and sensorimotor cognition. For instance, principles of causal motion (e.g., gravity and torque) relied upon the left IPS and left MTG, regions associated with perceiving and visualizing motion; when considering periodicity (e.g., wavelength frequency) activation was seen in regions associated with biorhythms (e.g., dancing, rhythm and meter in music, etc.) and terrestrial cycles (e.g., tidal patterns), including the dorsal PMC, bilateral parietal, and somatosensory cortex. These data suggest that, concurring with theories of grounded cognition, abstract scientific concepts are comprehended based on embodied visuospatial representations mapped onto corresponding cortical structures. Furthermore, these maps, grounded in sensorimotor codes, are distributed and comprised of features originating in disparate regions of the cortex, which suggests that a higher order organizational system is needed to assemble bottom-up features into a coherent conceptual representation—in other words, these data support the predictive coding theory of cortical organization.

### STEM learning interventions based in grounded cognition

Concepts in STEM learning range from those that can be readily experienced—e.g., if I jump, gravity forces me back to the ground—to ideas derived completely from mathematical equations, such as the enigmatic force known as dark matter. Given the accounts of abstract knowledge representations described above, we predict that concepts taught in STEM classrooms are better understood when they are initially grounded in hands-on learning activities. Given the nature of abstract concepts—variability across contexts (Granito et al., 2015) and the reliance on situational information (Barsalou & Weimer-Hastings, 2005)—grounding scientific and mathematical concepts in sensorimotor representations provides students with a useful tool for placing abstractions in a readily accessible, concrete conceptual framework. For instance, college students learning about angular momentum, the physical force keeping moving objects on a steady trajectory, can physically experience the concept by manipulating an apparatus on which this force was exerted (e.g., a bicycle wheel spinning on an axle held by the student). In a study that examined the advantages of using such a hands-on demonstration, students who actively engaged in the task demonstrated a greater understanding of the concept relative to students who had learned the same concept by merely observing the demonstration (Kontra et al., 2015). Additionally, fMRI data from the same participants demonstrated robust activation differences between the hands-on learning group and the observation group. Regions in the premotor, motor, sensory, and parietal cortex were more active when the active group answered questions about angular momentum. Moreover, the authors performed a mediation analysis to demonstrate that the activation in the primary motor cortex accounted for the between-group performance difference on the test of concept understanding (Kontra et al., 2015). According to embodied theories rooted in Hebbian learning and predictive coding, these results are a consequence of multimodal (kinesthetic, visual, proprioceptive, affective, etc.) associations integrated into a high-level conceptual representation at the time of learning. In other words, the students in the hands-on group are able to retrieve a rich, sensorimotor representation, which in turn facilitates their understanding of the abstract concept of angular momentum, as evidenced by the neural and behavioral data.

A number of studies (e.g., Brooks, Ouh-Young, Battert, & Kilpatrich, 1990; Han & Black, 2011) have observed similar results. Han and Black used virtual learning environments, enabling elementary students to develop visual, auditory, and haptic representations of mechanical principles. The authors concluded that the addition of the haptic dimension improved students’ performance. While these data concur with the idea that contextual features are absent in abstract representations, they suggest an important role for the hands-on experience. Perhaps, as proposed by radical embodied theories (e.g., Wilson & Golonka, 2013), motorically acting upon the world confers knowledge otherwise unavailable. Another plausible hypothesis is an extension of Dehaene and Cohen’s (2007) theory of neuronal recycling. Processes such as arithmetic and writing co-opt information processing mechanisms in regions of the cortex evolutionarily optimized for motor and spatial functions, such as bilateral IPS and left OTC, taking advantage of abilities, such as numerical quantity differentiation (e.g., three bananas is more than one banana) seen in monkeys, rats, and other altricial species. Thus, the concept of numerical magnitude is understood using the mechanisms of the visual/haptic system that evolved to process magnitude in a physical sense (e.g., estimating distance to an object one intends to grasp). In a similar vein, haptic experiences may enable human beings to represent concepts in motor cortex as described above.

In addition to hands-on activities, traditional learning materials (e.g., schematic pictures and symbol representations) are effective insofar as they are easily generalizable, and thus must be included in the STEM curriculum (Fyfe, McNeil, Son, & Goldstone, 2014). A curriculum which begins by teaching concepts via hands-on, concrete activities before moving into more abstract materials may be best suited to teach complex scientific and mathematical information. Fyfe and colleagues propose a curriculum that orients students with concepts by first using hands-on techniques and then gradually moving to abstract materials traditionally associated with mathematics and science—e.g., equations and illustrative models. In other words, when initially learning a concept, it is useful to constrain knowledge to sensorimotor referents before placing it in a context divorced from one’s first-hand experiences. In such a learning framework, confusing abstract ideas are first related to familiar, concrete objects, which aids in recall when ambiguous abstract symbols are insufficient—eventually, however, it is beneficial to strip such concepts down to their fundamental core. However, as evidenced by prior research (e.g., Barsalou & Weimer-Hastings, 2005; Binder et al., 2005), abstract concepts may eventually become left lateralized and generalizable, providing students with a neural scaffolding and enabling conventional teaching methods to be more easily understood. It is therefore important that students learn to apply such concepts independently of the context in which they were initially learned, allowing them to easily generalize across disciplines (e.g., geometry to physics). This sort of scaffolding may attenuate the variability in the effectiveness of laboratory-based activities, which can often be attributed to confusing, overwhelming, or boring laboratory procedures which have vague or ambiguous connections to the concepts they are intended to convey (Kirschner, Sweller, & Clark, 2006; Prince, 2004).

There have been conflicting reports concerning the benefits of haptic experience on learning outcomes, as some studies (e.g., Reiner, 1999) suggest that physical sensation itself improves learning, while others (e.g., Klahr, Triona, & Williams, 2007; Triona & Klahr, 2003; Olympiou & Zachariah, 2012) dispute this notion, demonstrating that activities performed in virtual learning environments result in similar benefits. In this vein, Klahr and colleagues (Klahr et al., 2007; Triona & Klahr, 2003) and Olympiou & Zachariah (2012) argue that the degree to which learners are actively engaged in the learning process—rather than physical activity per se—determines the outcome of learning. Accordingly, virtual learning, they argue, can benefit students as much as physical laboratory-based learning when it comes to conceptual understanding. In other words, virtual activities may improve students’ understanding of conceptual knowledge as much as physical activities—instead the relevant variable is whether the students can actively manipulate the materials (virtual or physical) in the process of learning. However, in these examples the virtual laboratories use components that strongly resemble familiar physical materials (e.g., glass beakers and digital thermometers used to study changes in temperature of various materials). In other words, these virtual laboratories may already be somewhat grounded in physical experience. It remains unknown, therefore, to what degree the learning gains in the virtual task rely on the neural representations of familiar physical representations, which would not be observed for laboratories that involve unfamiliar materials. Similarly, the targeted concepts in these studies (e.g., heat transfer and the mechanical properties of springs) did not specifically produce phenomena that were surprising or counter-intuitive. It remains unknown whether learning in such situations would be facilitated or impeded by experiencing only a virtual simulation versus a hands-on laboratory.

### Suggestions for using neuroimaging to study STEM learning

One potential way of understanding how the human brain learns complex scientific concepts is to examine learning outcomes based on neural markers (e.g., Cross et al., 2009; Davachi, Maril & Wagner, 2001; Kontra et al., 2015; Mason & Just, 2016) in addition to behavioral measures. The studies described above that reveal the neural basis of abstract concept representations in physics (Kontra et al., 2015; Mason & Just, 2016) provide neural markers for conceptual learning that can be used in addition to more traditional paper-and-pencil tests of learning. What remains to be seen is whether combining the neural data with the traditional tests adds unique explanatory power in predicting which students will retain their conceptual understanding and be able to use it appropriately at a later time, say 6 months or a year later. For example, future studies can take an active learning paradigm such as the one used by Kontra et al. (2015) for teaching about the concept of angular momentum and follow up with both a unit test from a textbook, as well as a computerized task that taps conceptual understanding while students undergo fMRI to record brain activity. Then students can return to the laboratory at some later time (e.g., after 6 months) for another paper-and-pencil test on the same topic, or even for a behavioral test of knowledge retention and transfer; for example, a demonstration of conceptual knowledge in which the student makes predictions about the outcome of a laboratory experiment. The key question would be whether performance on the follow-up test would be best predicted by the original paper-and-pencil test, or by the fMRI data, or by both combined. Such a demonstration would not only confirm that we have successfully identified sensitive neural markers of conceptual learning, it would also open the door for the use of fMRI in conjunction with traditional methods when testing the efficacy of a new instructional approach or laboratory procedure.

Another challenge in teaching students to identify abstract concepts from observable scientific data is that, by definition, abstract concepts are not consistently associated with invariable observable physical features across different contexts. Therefore, we must continue investigating the influence of context on abstract conceptual representation. One way of doing this is by directly comparing neural activity across ambiguous descriptions of object concepts when different contexts are provided. For instance, evoking a context by giving instructions to point out objects fitting a given description—e.g., a functional unit positioned at the end of a support arm—might result in similar neural patterns when identifying functionally dissimilar objects—e.g., a hammer and a street light. Such comparisons made across categories, including functional, visual, and haptic similarity, could inform the current debate between amodal and sensorimotor theorists. Thus, if a hammer and a light pole are represented similarly in the cortex while individuals focus on the visual features of the objects, but differently while they focus on functional characteristics, it would be clear that contextual cues influence conceptual knowledge. Accordingly, we would expect to see distinct activation patterns when these principles are applied to abstract versus concrete concepts (Borghi & Cimatti, 2009; Granito et al., 2015). Asking a participant to determine the likely air temperature, for instance, would lead an observer to direct their gaze towards a scene as a whole searching for key indicators, which could be measured via eye tracking; conversely, evaluating the structural integrity of an engineering apparatus (e.g., a truss) might lead one to focus his or her gaze on specific features within the object itself (e.g., joint fixtures). Further, it is possible to infer which objects in a scene are important for understanding a given concept and at what point attention is directed towards a given object or the scene as a whole, for example, using multi-voxel pattern analysis (MVPA), in addition to eye tracking and correlated with measures of behavioral performance across subjects. This type of analysis can also examine the potential facilitating effect of top-down instructions (e.g., search cues) provided at various points in the instruction process. Research on multimedia instruction has identified several best practices for reducing cognitive load as well as pitfalls to avoid that lead to cognitive overload (Mayer & Moreno, 2003). The addition of neural markers of attention and of conceptual comprehension will aid in further determining when a student is attending the appropriate details of the lesson. This information can then be used to modify instruction accordingly, adjusting factors such as when and how to introduce new facts and visual details in a multimedia lesson. In this way, we will have an opportunity to design new methods of teaching STEM concepts, taking advantage of our understanding of the neural basis of conceptual understanding.

Finally, future work should also aim to understand the importance for conceptual learning of individual differences in specific cognitive abilities (e.g., verbal and visual working memory; spatial visualization ability), prior knowledge (e.g., earlier classes in the same content domain), and habits of thought (e.g., visual versus verbal cognitive style). Each of these factors may play an important role in determining which students are likely to adopt an effective learning approach in the context of a specific lesson, or who may need additional support to fully comprehend the new material. As noted above, cognitive overload is likely to occur when working memory capacity is surpassed in a given modality (Mayer & Moreno, 2003), such as when text and pictures appear onscreen simultaneously. One’s threshold for information overload that impairs task performance is dependent on the individual’s working memory capacity (Unsworth & Engle, 2007), and this capacity is also known to be somewhat separable across verbal versus visuospatial domains (Shah & Miyake, 1996). Thus, this type of cognitive ability difference can lead to variability in which students will most effectively learn from a specific lesson. Moreover, neural indices of working memory demand (e.g., Barber, Caffo, Pekar, & Mostofsky, 2013) can further refine our ability to detect cognitive overload as it is occurring during a specific task or instructional lesson, allowing for a careful analysis of the contribution of this individual difference factor to successful learning.

Similarly, individual differences in domains of cognitive ability are well established (Carroll & Maxwell, 1979; Cattell, 1963; Horn & Cattell, 1966) and correlate with performance in academic domains (Deary, Strand, Smith, & Fernandes, 2007; Shah & Miyake, 1996; Wai, Lubinski, & Benbow, 2005, 2009). Of particular interest to the current discussion, spatial abilities predict performance in STEM domains (Wai et al., 2005, 2009). Surprisingly, little research has investigated whether there are benefits to differentiating performance or study strategies based on measures of visual, verbal, and spatial *abilities*, which is an intriguing area of focus for future work. Instead of considering these domain-specific cognitive abilities, much attention has been given to ideas such as Gardner’s theory of Multiple Intelligences (Gardner, 1993), and the related idea of visual and verbal learning styles, in which self-described “visual learners” would prefer to learn from pictures rather than words, and “verbal learners” would prefer words over pictures. However, to date, no evidence exists to support these theories of preferences in terms of improving learning outcomes based on individuating instruction in this way (Pashler, McDaniel, Rohrer, & Bjork, 2008; Visser, Ashton, & Vernon, 2006). On the other hand, there does seem to be some support for self-report measures of cognitive style—consistencies in how an individual processes information across contexts (e.g., Kozhevnikov, Hegarty, & Mayer, 2002; Messick, 1984)—which correlate with verbal, spatial, and object (visual but non-spatial) domains (for a review, see Kozhevnikov, 2007). These dimensions of cognitive style correlate with some measures of ability (Blazhenkova & Kozhevnikov, 2009; Kozhevnikov, Kosslyn, & Shephard, 2005) as well as choice of career; for example, engineering majors are likely to rate more highly on the spatial visual style dimension, whereas artists are more likely to rate highly on the object visual style dimension. However, it is unclear to what degree cognitive styles overlap with cognitive abilities or whether they represent consistent but flexible task approaches or strategies that can affect task performance, but are somewhat malleable or amenable to changes in instructions.

In this vein, work in our laboratory has demonstrated distinct neural signatures for habits of thought corresponding to representing information in a verbal versus a visual modality (Hsu, Kraemer, Oliver, Schlichting, & Thompson-Schill, 2011; Kraemer, Hamilton, Messing, DeSantis, & Thompson-Schill, 2014a, Kraemer, Rosenberg, & Thompson-Schill, 2009). These propensities for task strategies (i.e., verbal and visual cognitive styles) have been shown to correspond to which types of information participants encode when only visual information is presented during a task (Kraemer et al., 2016). These individual differences in cognitive style have also been shown to correlate with what type of information is successfully encoded and recalled (Kraemer et al., 2016), performance on a visual feature retrieval task (Hsu et al., 2011), and decisions made by participants in ambiguous situations (Amit & Greene, 2012). Importantly, two of these studies have revealed that these task approaches are somewhat flexible given changes in task instructions (Kraemer et al., 2016) and task context (Hsu et al., 2011), indicating that these individual differences represent malleable factors that can potentially be leveraged to improve cognitive processing in a given context. At this point, more research is needed to determine whether and how these differences impact STEM learning specifically.

## Conclusions

Embodied theories of cognition have reshaped the landscape of cognitive science, providing a rich literature that has not only changed the way we look at the human mind, but also inspired innovative learning interventions. As American students continue to struggle in the STEM fields, it is imperative that scientists search for novel ways to improve the scientific pedagogy. Here, we propose that embodied exercises improve STEM learning by situating abstract concepts in a concrete context, thus correlating intangible ideas with corporeal information. In doing so, rich multimodal distributed neural representations are forged, giving students a better chance at succeeding in the sciences.
